# Whey proteins revisited: from an amino acid source to therapeutic potential—a narrative review

**DOI:** 10.3389/fnut.2026.1933363

**Published:** 2026-10-23

**Authors:** Mirosław Perliński, Sandra Banasiak, Karolina Tenderenda

**Affiliations:** Faculty of Pharmacy, University of VIZJA, Warsaw, Poland

**Keywords:** bioactive peptides, clinical nutrition, food special medical purpose, muscle metabolism, sarcopenia, whey proteins

## Abstract

**Background and aims:**

Whey proteins are well-established sources of indispensable amino acids, but their relevance to clinical nutrition may also depend on rapid digestion, leucine availability, sulfur-containing amino acids, and bioactive fractions. Previous reviews have commonly focused on sports nutrition, muscle protein synthesis, individual metabolic outcomes, or food-derived peptides, with less emphasis on how these characteristics may inform the formulation of Foods for Special Medical Purposes (FSMPs).

**Methods:**

This narrative review integrates clinical, preclinical, and mechanistic literature identified in PubMed, Scopus, and Web of Science, with emphasis on muscle metabolism, glycemic regulation, antioxidant and immunomodulatory pathways, wound healing, gut microbiota, and experimental protein-lipid complexes.

**Results:**

The most consistent human evidence supports rapid postprandial aminoacidemia, stimulation of muscle protein synthesis, and acute modulation of postprandial glycemia. Evidence for improvements in muscle-related outcomes is also available for selected older or malnourished populations, although whey protein has frequently been administered as part of a multinutrient formulation. Antioxidant, immunomodulatory, wound-healing, microbiota-related, and anticancer-related effects remain supported mainly by mechanistic, *in vitro*, animal, or limited clinical evidence.

**Conclusion:**

Whey proteins should be considered high-quality protein sources with potentially relevant biofunctional properties rather than established therapeutic agents. Their use in FSMPs should be tailored to the target population, required protein and leucine delivery, tolerance, processing characteristics, and overall formulation. Comparative randomized trials are needed to define formulation-specific effects, dose–response relationships, long-term efficacy, and safety.

## 1 Introduction

Enteral nutrition (EN) is widely recognized as the preferred method of nutritional support for patients requiring artificial feeding. Compared to parenteral nutrition, it is associated with reduced mortality, shorter hospital stays and lower healthcare costs. These benefits are largely attributed to the physiological route of nutrient delivery, which supports the integrity of the intestinal mucosa, helps prevent dysbiosis and bacterial translocation, and plays an important role in immune modulation (1).

Foods for Special Medical Purposes (FSMPs) are commonly used in enteral nutrition and are strictly regulated in terms of composition and safety. However, their clinical effectiveness depends not only on appropriate administration and monitoring, but also on the quality and type of nutrients used—particularly protein. As a macronutrient, protein is fundamental to numerous physiological processes including structural support, regulation, transport and immune function (2). It is essential for tissue repair, muscle preservation, hormone and enzyme synthesis, and immune defense (3). Therefore, both the quantity and quality of protein included in FSMPs should be carefully considered, especially in patients for whom these products represent the sole source of nutrition.

Various protein sources are used in medical nutrition products, including both plant- and animal-based. The nutritional quality of protein depends largely on its amino acid profile and digestibility. An optimal protein source is one that provides all essential amino acids in adequate proportions to effectively support protein synthesis in muscles, organs and other tissues (4). In this context, the presence of branched-chain amino acids (BCAAs), particularly leucine, is considered a key factor in anabolic stimulation (5).

Compared to soy proteins or casein, cow’s milk proteins exhibit a higher Digestible Indispensable Amino Acid Score (DIAAS; Figure 1), which is currently recommended by the Food and Agriculture Organization (FAO) as a method for evaluating protein quality. This approach accounts for the true ileal digestibility of each indispensable amino acid (6). Therefore, awareness of the protein source in commercially available formulas is of particular relevance, as it may influence not only dietary tolerance but also the rate of absorption and the efficiency of protein utilization (7, 8).

**Figure 1:**
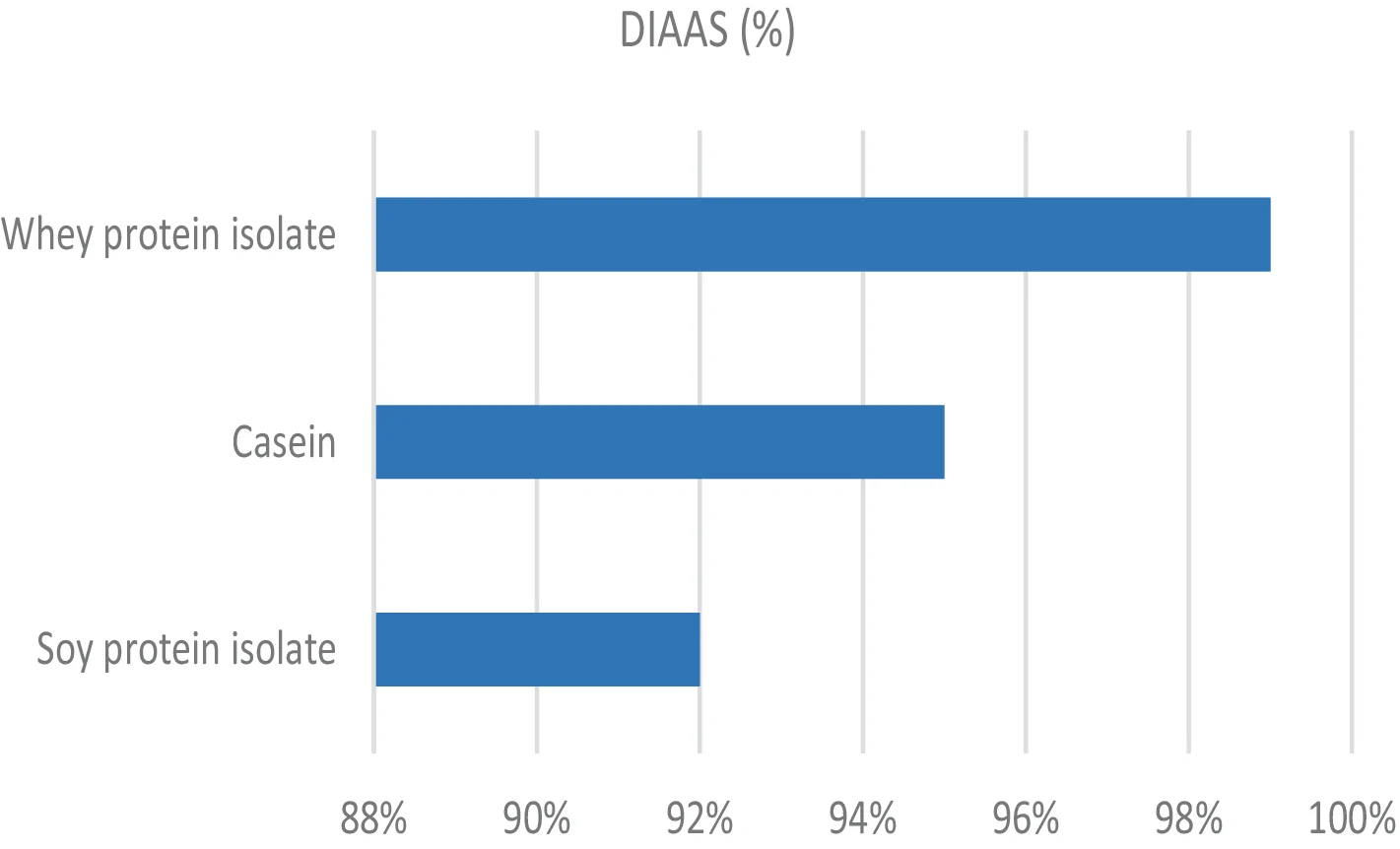
Reported digestible indispensable amino acid score values for whey protein isolate, casein, and soy protein isolate. Values reproduced from Savino (2) and were not independently calculated by the authors. The values correspond to the reference age group and original data source reported in Savino (2).

Despite well-established nutritional and functional advantages of cow’s milk proteins, soy protein isolates are widely used in the production of FSMPs due to their favorable cost, partly resulting from the high protein yield obtained from soybeans. It is estimated that the production cost of soy protein is approximately five times lower than that of whey proteins, which may explain differences observed between FSMPs products based on various protein sources. Nevertheless, soy-based formulas remain a valuable alternative for patients with cow’s milk protein allergy requiring enteral nutrition. Soybeans contain two major storage proteins-β-conglycinin and glycinin—which together account for approximately 65–89% of total soy protein content and are classified as globulins (9). Compared to whey or casein proteins, soy protein is inferior in certain aspects, particularly in terms of methionine content, which is lower than the reference pattern established by FAO and World Health Organization (WHO) experts (10, 11).

In recent years, increasing attention has been paid to the potential biofunctional properties of WPs. These include their effects on glycemic response (12), wound healing, muscle anabolism (3), antioxidant defense (13), immune modulation (3) or sarcopenia. Beyond being a source of high-quality amino acids, WPs contain peptides and metabolites that may exert direct physiological actions. Given their multifunctional profile, whey proteins are increasingly recognized as a valuable component in therapeutic nutrition (14).

Literature searches were performed in PubMed, Scopus, and Web of Science using a combination of MeSH terms and keywords such as “whey protein,” “bioactive peptides,” “muscle metabolism,” “clinical nutrition” and “sarcopenia.” Articles were selected based on predefined inclusion and exclusion criteria, with a focus on human and relevant animal studies examining the functional effects of whey proteins. Searches were limited to peer-reviewed articles published between 2000 and 2025 in English or Polish. A total of 289 articles were identified through electronic database searches. After removal of 43 duplicates, 246 records remained for title and abstract screening. Of these, 176 records were excluded because they were not relevant to the scope of the review. This remains 70 records for further assessment. An additional 12 relevant articles were identified through manual screening of reference lists, resulting in total of 82 articles assessed at the full-text stage. Studies meeting the predefined eligibility criteria were subsequently included in the narrative synthesis.

Inclusion criteria:

- Studies investigating the composition, metabolism, or physiological effects of WPs.
- Research involving human subjects or animal models relevant to clinical nutrition.
- Articles discussing the role of whey proteins in enteral or therapeutic nutrition.
- Peer-reviewed original research articles, reviews, or clinical trials.

Exclusion criteria:

- Studies unrelated to nutritional or functional outcomes of whey proteins.
- Non-peer-reviewed sources (e.g., conference abstracts, editorials, gray literature).
- Publications focused exclusively on sports nutrition or exercise physiology witout a clinical context.
- Articles with insufficient methodological details or lacking relevance to FSMPs.

The literature was thematically synthesized according to whey protein composition and nutritional quality, bioactive peptides and physiological mechanisms, effects on muscle metabolism, metabolic and gastrointestinal effects, clinical applications, and potential clinical relevance. Given the narrative nature of this review and the heterogeneity of the available evidence, no formal risk-of-bias or certainty-of evidence assessment was performed. However, the level and type of evidence were considered when interpreting the findings. Evidence from randomized controlled trials and other human clinical studies was distinguished from findings derived from observational studies, animal model, and *in vitro* or mechanistic investigations. Clinical implications were primarily based on human evidence, whereas preclinical and mechanistic findings were used to provide biological context and to explore potential mechanisms. Findings based predominantly on preclinical evidence were therefore interpreted cautiously and were not considered to constitute established clinical effects. Due to substantial heterogeneity in study populations, whey protein preparations, interventions, outcomes, and study designs, no quantitative meta-analysis was performed. There is also a lack of large, randomized controlled trials (RCTs) in certain clinical indications, as well as consideration in studies of the variability in forms of whey protein (isolate, concentrate, hydrolysate) and dosing, particularly intake per meal versus total daily consumption. The reviewed literature consistently supports the classification of WPs not only as a high-quality amino acid source, but as a biofunctional agent with diverse physiological benefits.

Previous reviews have established the nutritional quality of whey proteins and have examined selected outcomes such as muscle protein synthesis, metabolic regulation, or food-derived bioactive peptides. The present review does not seek to redefine these established properties. Its specific contribution is to integrate evidence on whey protein composition, processing form, digestion kinetics, bioactive fractions, and level of clinical validation within an FSMP-oriented framework. Particular emphasis is placed on distinguishing clinically demonstrated effects from mechanistic plausibility and on identifying how population characteristics, dose, timing, co-formulated nutrients, and protein form may influence translational relevance (Table 1).

**Table 1:** Summary of selected studies and mechanistic evidence regarding the biofunctional properties of whey proteins.

Area	Study/evidence type	Model/population	Whey-related intervention or component	Main findings	Translational interpretation
Muscle protein synthesis	Human clinical / experimental studies	Young and older adults	Whey protein isolate or hydrolysate compared with casein or soy	Whey proteins were associated with stronger postprandial stimulation of muscle protein synthesis, largely attributed to rapid digestion and high leucine availability.	Human evidence supports the role of whey proteins in stimulating MPS, particularly in older adults or individuals at risk of anabolic resistance.
Sarcopenia and functional outcomes	Clinical studies / RCT-based evidence	Older adults with sarcopenia or risk of muscle loss	Whey protein enriched with leucine and vitamin D	Supplementation was associated with improvements in muscle-related outcomes in selected populations.	Clinical relevance is strongest when whey protein is combined with other anabolic nutrients, but optimal dosing and patient selection remain to be clarified.
Glycemic regulation	Human clinical studies and mechanistic studies	Healthy individuals, individuals with impaired glucose tolerance or T2DM	Whey protein preload or whey-containing meal	Whey protein intake was associated with increased incretin response, enhanced insulin secretion, and reduced postprandial glycemic excursions.	Human evidence supports acute metabolic effects, although long-term clinical benefits require further confirmation.
DPP-IV inhibition	*In vitro* / mechanistic studies	Enzymatic and peptide-based models	Whey-derived peptides, especially hydrolysates	Certain whey peptides showed DPP-IV inhibitory activity, potentially prolonging incretin action.	Mechanistic plausibility is strong, but direct confirmation of clinically meaningful DPP-IV inhibition in humans remains limited.
Antioxidant and immunomodulatory effects	Preclinical and selected clinical evidence	Cell models, animal studies, and selected human populations	Cysteine-rich whey proteins and bioactive fractions	Whey proteins may support glutathione synthesis and modulate inflammatory mediators.	Evidence supports biological plausibility, but clinical effects vary by population, dose, and formulation.
Wound healing	Preclinical and limited clinical evidence	Experimental wound models and nutritionally vulnerable patients	Whey protein and hydrolyzed whey fractions	Whey proteins may support collagen synthesis, antioxidant defense, and tissue repair processes.	Potentially relevant in malnourished, elderly, or post-surgical patients, but dedicated clinical trials are still needed.
Anticancer-related mechanisms	Primarily in vitro and animal studies	Cancer cell lines and animal tumor models	HAMLET, BAMLET and related protein–lipid complexes	Selective cytotoxicity against cancer cells has been reported in experimental models.	Findings are promising but should not be interpreted as established clinical anticancer effects in humans.
Gut microbiota modulation	Preclinical and limited clinical evidence	Animal models and selected human-related data	Whey proteins and hydrolysates	Whey proteins may influence beneficial bacterial genera and short-chain fatty acids production.	This remains an emerging area with limited clinical validation.

## 2 Muscle protein synthesis and sarcopenia prevention

Whey proteins are widely regarded as one of the most effective dietary proteins for stimulating muscle protein synthesis (MPS). Their rapid digestibility, high biological value, and rich content of BCAAs—particularly leucine—make them especially well-suited for supporting the maintenance of muscle mass, particularly in catabolic states such as aging, immobilization, malnutrition, and illness (15).

### 2.1 Mechanistic basis

Leucine acts as a key nutrient signal that stimulates MPS primarily through activation of the mammalian target of rapamycin complex 1 (mTORC1) pathway. WPs contain approximately 14% leucine, making them one of the richest known protein sources of this key anabolic amino acid (16, 17).

In contrast to casein, which forms gastric clots and is digested more slowly, WPs are rapidly hydrolyzed and absorbed in the small intestine. This results in a rapid and pronounced postprandial increase in plasma amino acid concentrations, which is closely associated with a strong stimulation of muscle protein synthesis (8, 17).

### 2.2 Evidence from experimental and human studies

Accumulating evidence indicates that specific whey protein preparations, including whey protein isolate, may stimulate muscle protein synthesis more effectively than other commonly used protein sources, such as casein or soy, under selected experimental conditions (8, 17, 18). These findings have been attributed in part to the rapid availability of amino acids and the high leucine content of whey protein preparations.

This effect appears to be particularly relevant in older adults, in whom anabolic resistance reduces the efficiency of muscle protein synthesis. In this context, whey proteins have been shown to enhance postprandial muscle protein accretion more effectively than slower-digesting proteins (8, 18).

Taken together, these observations emphasize that not only the quantity but also the quality, composition and timing of protein intake are critical determinants of muscle protein metabolism, especially in populations at risk of sarcopenia.

### 2.3 Clinical application in sarcopenia and FSMPs

Given the anabolic properties, whey proteins have been increasingly incorporated into FSMPs designed for older adults or patients at risk of muscle wasting.

Clinical data suggest that whey protein-containing formulations may improve muscle strength and functional outcomes in selected individuals, particularly when whey protein is provided as part of multi-nutrient formulation containing leucine and vitamin D (19). However, in such combined interventions, the observed effects cannot be attributed to whey protein alone, and the findings should be interpreted as evidence for the potential benefit of the overall nutrition formulation. In contrast, finding from interventions in which whey protein is administered without additional anabolic nutrients provide more direct evidence regarding the effects attributable to whey protein itself.

In studies of older adults, per-meal protein doses in the range of approximately 25–30 g, providing approximately 2.5–3 g of leucine, have often been used or proposed to overcome anabolic resistance (20). These values should be interpreted as population- and context-dependent targets rather than universal FSMP dosing recommendations.

In addition, WP-enriched FSMPs have shown potential in supporting muscle preservation during intentional weight loss in obese older adults (21), as well as in enhancing recovery following surgery or hospitalization (22). The extent to which these effects are attributable specifically to whey protein may depend on the composition of the formulations and the presence of other nutritional components.

Notably, emerging evidence suggests that WPs may stimulate greater muscle anabolism than an equivalent amount of free essential amino acids, indicating that bioactive peptides and protein-derived micro fractions may contribute to their overall anabolic effect (7).

### 2.4 Broader implications

Beyond sarcopenia, whey protein-based interventions are increasingly being explored in other conditions associated with muscle catabolism, including cancer cachexia, critical illness and chronic inflammatory states. Their capacity to support functional recovery, mobility, and overall nutritional status further highlights their clinical relevance as a key component of muscle-targeted medical nutrition strategies (23–25).

## 3 Glycemic control and metabolic regulation

WPs have gained increasing scientific interest due to their ability to influence postprandial glycemic response and support glucose–insulin homeostasis, particularly in individuals with insulin resistance, prediabetes, or type 2 diabetes mellitus (T2DM). Their metabolic effects appear to be mediated through a combination of hormonal, digestive, and cellular mechanisms (26, 27).

### 3.1 Incretin hormone stimulation

One of the key mechanisms underlying the glycemic effects of WPs is the stimulation of incretin hormones, particularly glucagon-like peptide-1 (GLP-1) and glucose-dependent insulinotropic polypeptide (GIP). These hormones enhance postprandial insulin secretion, suppress glucagon release, and delay gastric emptying, thereby reducing the rate of glucose absorption (28, 29).

Evidence from clinical studies indicates that consumption of WPs—either as a preload or as part of a meal—is associated with increased circulating levels of GLP-1 and GIP, leading to enhanced insulin secretion and attenuated postprandial glucose excursions in both healthy individuals and patients with T2DM (29–31).

### 3.2 Rapid digestibility and amino acid response

Intact whey protein are generally rapidly digested and absorbed, resulting in a pronounced rise in plasma amino acid concentrations, especially BCAAs such as leucine, isoleucine, and valine. These amino acids contribute to the stimulation of pancreatic insulin secretion, both directly and through incretin-mediated pathways (32–34).

Leucine, in particular, activates mTOR signaling in pancreatic β-cells, thereby promoting glucose-stimulated insulin secretion (GSIS). Consistent findings indicate that WP preloads can reduce the area under the glucose curve (AUC) and improve early-phase insulin response following carbohydrate intake (29, 30).

### 3.3 DPP-IV inhibition

Another mechanism that may contribute to the glycemic effects of WPs involves inhibition of dipeptidyl peptidase IV (DPP-IV), an enzyme responsible for the rapid degradation of incretin hormones. Peptides generated from hydrolyzed whey protein, including β-lactoglobulin-rich whey protein concentrates, have demonstrated DPP-IV inhibitory activity *in vitro*, potentially prolonging incretin action and improving glucose tolerance (28, 35, 36). These findings suggest a potential mechanism by which whey-derived peptides could influence incretin-mediated glucose regulation; however, the inhibitory activity observed *in vitro* should not be interpreted as evidence of a clinically meaningful improvement of glycemic control. Notably, DPP-IV inhibitory activity appears to be more pronounced in hydrolyzed forms of whey protein than in native preparations. Thus, the potential contribution of DPP-IV inhibition to the metabolic effects of WPs remains primarily mechanistic and requires confirmation in well-controlled human studies.

### 3.4 Clinical implications and human trials

Accumulating clinical evidence supports the beneficial effects of WPs on postprandial glycemia. Available data suggest that WP supplementation may reduce postprandial blood glucose levels, particularly through delayed gastric emptying and enhanced incretin responses (30, 31). Similar effects have been observed in individuals with impaired glucose tolerance, where whey protein intake has been associated with improved glycemic control and additional cardiometabolic benefits (37). Furthermore, WPs appear to enhance GSIS through both amino acid-dependent mechanisms and incretin-mediated pathways (12). These properties are particularly relevant in the context of medical nutrition products, where WP-based formulations may support glycemic management in patients with diabetes, metabolic syndrome, or obesity.

## 4 Antioxidant and immunomodulatory effects

Whey proteins exert significant antioxidant and immunomodulatory effects, largely attributable to their amino acid composition and the presence of bioactive peptides. A key component in this context is cysteine, which serves as a precursor for glutathione (GSH)—the most abundant intracellular antioxidant, essential for maintaining redox homeostasis and protection against oxidative stress (38, 39).

The sulfhydryl (-SH) group of cysteine acts as a potent reducing agent, enabling GSH to neutralize reactive oxygen species (ROS) and maintain cellular thiol-disulfide balance. Compared to other dietary proteins, such as casein or soy, whey proteins contain higher levels of sulfur-containing amino acids essential for GSH biosynthesis (32, 38). *In vitro* findings further support these observations, demonstrating cytoprotective effects of why protein isolate through enhanced intracellular antioxidant capacity (38).

Available evidence for both clinical and preclinical studies indicates that whey protein supplementation may increase GSH levels and improve oxidative status in various populations. This has been associated with reductions in pro-inflammatory cytokines including IL-1β.

IL-6 and TNF-α, alongside increased levels of anti-inflammatory mediators such as IL-10 (1, 37, 39). *In vitro* findings further support these observations, demonstrating cytoprotective effects of whey protein through enhanced intracellular antioxidant capacity (38).

Collectively, these findings suggest that WPs may enhance immune function by modulating inflammatory responses, supporting innate immunity and improving cellular resilience under conditions of metabolic stress, such as aging, obesity or chronic illness.

Additionally, bioactive whey-derived components, including lactoferrin and β-lactoglobulin fragments, have been shown to exert direct immunomodulatory effects, such as antimicrobial activity, modulation of phagocyte function, and regulation of inflammatory signaling pathways (14, 32). Due to these properties, whey protein could potentially be utilized in future research involving oncology-related diseases or other conditions associated with high levels of inflammation in patients.

Whey bioactivity cannot be attributed to a single peptide class. β-Lactoglobulin and α-lactalbumin are precursors of encrypted peptide sequences released during gastrointestinal or enzymatic hydrolysis, whereas lactoferrin is a bioactive intact protein that can also generate antimicrobial fragments. Peptides generated from β-lactoglobulin-rich preparations have demonstrated DPP-IV inhibitory activity in enzymatic assays, providing a plausible mechanism for prolonged incretin action. Lactoferrin and lactoferrin-derived fragments may interact with microbial membranes and host immune pathways, whereas cysteine-rich whey fractions may indirectly support antioxidant defence by increasing substrate availability for glutathione synthesis. Opioid-like whey-derived peptides have also been described, but their gastrointestinal stability and clinical relevance remain uncertain. Importantly, the detection of activity in an enzyme or cell assay does not establish that an effective peptide concentration is achieved in human plasma or target tissues after oral ingestion.

## 5 Wound healing and tissue regeneration

Wound healing is a complex, energy- and nutrient-dependent process involving inflammation, cellular proliferation, angiogenesis, collagen synthesis, and tissue remodeling. Adequate protein intake—particularly from high-quality sources—is essential for optimal healing, especially in malnourished, post-operative, or elderly patients.

Due to their rapid digestibility and favorable amino acid profile, whey proteins play an important role in supporting tissue repair and regeneration.

### 5.1 Amino acid composition supporting tissue repair

WPs provide several amino acids essential for wound healing and collagen synthesis. Glycine and proline serve as key structural components of collagen, the primary protein of the extracellular matrix. Arginine contributes to nitric oxide production, thereby supporting angiogenesis and local blood flow. BCAAs, particularly leucine, are involved in anabolic signaling and cellular proliferation, while cysteine supports glutathione synthesis and local antioxidant defense.

This amino acid profile supports the use of WPs as a targeted nutritional strategy in wound management, particularly in patients receiving enteral nutrition.

### 5.2 Experimental evidence

Experimental and preclinical data suggest that whey protein supplementation may accelerate wound healing process. Observed effects include reduced inflammatory infiltration, modulation of cytokine profiles, and enhanced formation of granulation tissue and re-epithelialization (31, 37). Hydrolyzed forms of whey protein may further enhance these effects due to faster amino acid availability and improved support of collagen synthesis and angiogenic signaling.

### 5.3 Clinical relevance

In clinical practice, impaired wound healing is commonly observed in older adults, patients with pressure ulcers or diabetic wounds, and those recovering from surgery. In these populations, whey protein- enriched oral nutritional supplements (ONS) and FSMPs may help address protein deficits while delivering key amino acids that support tissue repair.

In addition to providing structural substrates, the antioxidant and immunomodulatory properties of WPs may further facilitate wound healing by modulating inflammation, reducing oxidative damage, and supporting immune function during tissue repair.

## 6 Experimental protein-lipid complexes and emerging microbiota research

Among the most intriguing findings in this area are whey protein-derived complexes that exhibit selective cytotoxicity against cancer cells. Notably, complexes such as HAMLET (Human Alpha-lactalbumin Made Lethal to Tumor Cells), formed by α-lactalbumin and oleic acid, have demonstrated tumor-selective cytotoxicity and apoptosis-inducing activity in both *in vitro* and *in vivo* models. HAMLET has been investigated across a range of tumor cell lines, including glioblastoma, bladder cancer and colon cancer, while showing limited effects on healthy, differentiated cells (40–42). Similar cytotoxic properties have been described for related complexes such as BAMLET (Bovine Alpha-lactalbumin Made Lethal to Tumor cells) and BLAGLET, derived from β-lactoglobulin. These complexes promote chromatin condensation, mitochondrial membrane depolarization, and activation of caspase-dependent apoptosis. Mechanistically, these protein-lipid complexes appear to penetrate tumor cell membranes and induce cell death through multiple pathways, including disruption of calcium homeostasis, mitochondrial dysfunction, generation of reactive oxygen species (ROS) and autophagic mechanisms (40, 41).

Evidence from animal models further supports their potential therapeutical effects, as oral administration of HAMLET has been associated with delayed tumor progression and improved survival without systemic toxicity (42).

It should be emphasized that the anticancer-related effects of whey-derived protein-lipid complexes are based predominantly on *in vitro* and animal studies. Although these findings provide valuable mechanistic insight and suggest potential directions for future research, they cannot currently be translated into clinical recommendations for cancer prevention or treatment. In the context of clinical nutrition, these observations should therefore be interpreted cautiously and regarded as experimental evidence rather than established therapeutic applicability.

### 6.1 Immunomodulatory and antioxidant synergy in cancer contexts

Beyond direct cytotoxic effects, whey proteins may contribute to cancer related defense mechanisms through immunomodulatory and antioxidant pathways. Their high cysteine content supports glutathione synthesis, which may protect normal cells from oxidative DNA damage while simultaneously immune functions. Available evidence suggests that WPs may increase natural killer cell activity, regulate T-cell proliferation, and attenuate chronic inflammatory signaling associated with tumor initiation and progression (32, 43, 44).

### 6.2 Modulation of gut microbiota and the gut–immune–brain Axis

In addition to their potential anticancer effects, WPs have been shown to influence gut microbiota composition, a key regulator of host metabolism, immunity, and disease susceptibility. Both clinical and preclinical studies indicate that WPs and their hydrolysates may promote the growth of beneficial bacterial genera, such as Bifidobacterium and Lactobacillus, while suppressing opportunistic pathogens (45). Furthermore, whey proteins appear to modulate the production of short-chain fatty acid, particularly butyrate, which is known for its anti-inflammatory and anti-proliferative properties. These effects may contribute to improved mucosal integrity and enhanced systemic immune competence through modulation of gut-immune axis (46).

Emerging evidence also suggests a potential influence on the gut-brain axis, as WP-induced alterations in microbiota composition have been associated with improved cognitive function and reduced anxiety-like behavior in preclinical models, although clinical validation remains limited.

## 7 Clinical relevance in FSMPs

Given their multifunctional properties, whey proteins incorporated into FSMP formulations may support selected nutritional or functional outcomes across a range of patient populations. Their use has been associated with enhanced recovery, potential reduction in hospital stays, and support for both and metabolic function.

In clinical practice, whey proteins may serve as a strategic component of nutritional therapy in conditions such as sarcopenia, diabetes, post-surgical recovery and malnutrition. Their favorable amino acid profile and biofunctional properties make them particularly suitable for patients with increased metabolic demands or impaired nutritional status.

Human metabolic studies indicate that whey protein preparations, including whey protein isolate and hydrolysate, can stimulate postprandial muscle protein synthesis more strongly than casein or soy under selected experimental conditions, an effect attributed to rapid digestion and leucine availability (7, 8, 17, 18). Evidence regarding longer-term functional outcomes is less uniform. In older adults with sarcopenia or at risk of muscle loss, improvements have been reported with formulations combining whey protein, additional leucine, and vitamin D (19–21, 25). Such findings support the clinical relevance of muscle-targeted FSMPs, but they do not establish that whey protein alone produced the observed effect. Benefits may depend on baseline protein intake, vitamin D status, degree of sarcopenia, intervention duration, physical activity, and adherence. In obese older adults undergoing intentional weight loss, a whey-, leucine-, and vitamin D-enriched supplement helped preserve muscle mass (21). These findings are population-specific and should not be extrapolated automatically to critically ill, renally impaired, or oncological patients.

Furthermore, available evidence suggests that whey protein-based formulations may offer advantages over plant-based or collagen-only protein sources, particularly in terms of anabolic potential and overall functional effect.

These observations support a shift in the perception of whey proteins as solely a nutritional component.

The incorporation of whey protein into an FSMP should begin with the clinical purpose of the product rather than an assumption that a higher whey proportion is universally superior. Relevant considerations include total protein and energy requirements; indispensable amino acid and leucine delivery per clinically feasible serving; the need for rapid versus sustained amino acid availability; renal, hepatic, and metabolic status; fluid-volume restrictions; gastrointestinal tolerance; milk protein allergy; and whether the product is intended for oral, bolus, or continuous enteral administration.

Whey concentrate, isolate, and hydrolysate are not interchangeable. They may differ in protein purity, residual lactose and fat, peptide profile, osmolality, taste, allergenic potential, digestion kinetics, processing stability, and cost. Hydrolysis may facilitate digestion in selected contexts but does not guarantee superior clinical effectiveness and may increase bitterness or alter osmolality. The contribution of co-formulated casein, plant protein, collagen, leucine, vitamin D, carbohydrate, fat, and fiber should also be considered. Product-specific clinical evidence should therefore be preferred over assumptions based solely on the name of the protein source.

## 8 Discussion

The available evidence supports a growing recognition of whey proteins as more than a source of essential amino acids, highlighting their role as multifunctional components of clinical nutrition.

A critical interpretation of the available evidence indicates that the biological effects of whey proteins differ substantially in terms of strength and type of supporting data. The most consistent human evidence relates to muscle protein synthesis and postprandial glycemic regulation, where rapid digestibility, high leucine content, and incretin-mediated pathways have been repeatedly implicated. In contrast, several other proposed effects, including DPP-IV inhibition by specific whey-derived peptides, microbiota modulation, and anticancer activity of protein-lipid complexes, are supported mainly by *in vitro* or preclinical studies. Therefore, while these mechanisms are biologically plausible and relevant for future research, they should not be interpreted as established clinical effects.

This distinction is particularly important for clinical nutrition and FSMP formulation. Whey proteins may provide functional advantages not only because of their indispensable amino acid profile, but also because of their peptide fractions, sulfur-containing amino acids, and rapid postprandial aminoacidemia. However, the magnitude of these effects depends on the form of whey protein used, including isolate, concentrate, or hydrolysate, as well as the dose, timing of administration, co-formulated nutrients, and the metabolic status of the target population. Consequently, evidence obtained in healthy adults, sports-related settings, or preclinical models cannot be directly generalized to malnourished, elderly, critically ill, or metabolically compromised patients.

Taken together, the current evidence supports a translational framework in which whey proteins should be viewed as multifunctional nutritional components with varying levels of evidence across clinical indications. Their role appears strongest in supporting muscle anabolism and acute metabolic regulation, while their immunomodulatory, microbiota-related, wound-healing, and anticancer-related effects require further confirmation in well-designed human studies.

Compared with other protein sources used in FSMPs—such as casein, soy, or collagen—whey proteins exhibit distinct functional advantages. Their rapid digestibility and absorption result in a swift postprandial rise in plasma amino acid concentrations, which is critical for effective anabolic signaling. In particular, their high content of branched-chain amino acids, especially leucine, plays a central role in the activation of muscle protein synthesis via the mTOR pathway. Additionally, whey proteins provide significant amounts of sulfur-containing amino acids, including cysteine and methionine, which serve as a precursor for glutathione, a key element of cellular antioxidant defense.

These properties translate into clinically relevant effects across a range of patient populations. In the context of sarcopenia and age-related muscle loss, WP-enriched nutritional strategies have been associated with improvements in muscle mass and functional performance. In metabolic disorders such as type 2 diabetes and prediabetes, whey proteins may contribute to improved glycemic control though mechanisms involving incretin stimulation and delayed gastric emptying. Similarly, in critically ill or post-operative patients, their immunomodulatory and antioxidant properties may support recovery processes and enhance host defense. These effects are particularly relevant in elderly and malnourished patients, where improved protein quality may contribute to better nitrogen tissue repair and overall functional status.

Notably, accumulating evidence suggests that whey protein may promote greater muscle protein accrual than an equivalent amount of free amino acids, indicating that matrix effects—such as the presence of bioactive peptides and associated compounds—play a significant role beyond amino acid composition alone.

Beyond intact proteins, bioactive peptides released during digestion or through enzymatic hydrolysis are likely responsible for many of the observed physiological effects. These include peptides with ACE-inhibitory activity, opioid-like peptides influencing gastrointestinal function and DPP-IV inhibitory peptides that may prolong incretin action and support glycemic regulation. In addition, components such as immunoglobulins and lactoferrin contribute to antimicrobial and immunoregulatory functions.

Emerging evidence from experimental models also point to the anticancer potential of protein-lipid complexes such as HAMLET and BAMLET, although their clinical relevance remains to be fully established.

Despite these promising findings, several limitations should be acknowledged. The heterogeneity of available studies limits direct comparisons and preclude robust quantitative synthesis. Furthermore, there is a lack of large-scale randomized controlled trials in certain clinical areas, including wound healing and oncology support. Variability in the form of whey protein (e.g., isolate, concentrate, hydrolysate), as well as differences in dosing strategies complicates the interpretation and generalization of results. Mechanisms related to gut microbiota modulation and neuroendocrine interactions also remain insufficiently explored. In addition, there is no clear consensus regarding optimal dosing regimens or the long-term safety of high-dose WP supplementation in specific patient populations. From practical and formulation perspective, these findings suggest that whey proteins should be considered a key component of FSMPs aimed at supporting muscle metabolism, metabolic regulation, and immune function. Their effectiveness may be further enhanced through combination with other functional ingredients such as leucine, vitamin D, or prebiotics to achieve synergistic effects. At the same time, careful formulation strategies are required to avoid dilution of their bio functional potential, particularly through excessive inclusion of lower-quality protein sources.

Overall, whey-protein based formulations should emphasize not only nutritional adequacy but also their broader therapeutic functionality, especially in populations with those with increased physiological demands.

Taken together, the available evidence supports a shift in the perception of whey proteins—from a purely nutritional substrate to a biologically active and clinically relevant component of modern therapeutic nutrition.

The effects of whey protein should not be treated as uniform across patient groups. Older adults with anabolic resistance may require a different per-meal stimulus from younger adults; acute glycemic effects observed after a whey preload in type 2 diabetes do not establish long-term disease modification; and findings from healthy volunteers or exercise studies cannot be extrapolated directly to critically ill, oncological, renally impaired, or severely malnourished patients.

### 8.1 Structure–activity relationships and mechanistic interpretation

The biofunctional properties of whey proteins are closely related to their structural and compositional characteristics. First, their high leucine content contributes to activation of anabolic signaling pathways, particularly mTORC1, which is central to muscle protein synthesis. Second, their rapid gastric emptying and intestinal absorption result in a rapid postprandial increase in circulating amino acids, which may partly explain their stronger anabolic response compared with slower-digesting proteins such as casein. Third, sulfur-containing amino acids, particularly cysteine, support glutathione synthesis and may therefore contribute to antioxidant defense and immune regulation.

In addition to intact proteins, whey-derived peptides released during digestion or enzymatic hydrolysis may exert specific biological activities. Hydrolyzed whey fractions may contain peptides with DPP-IV inhibitory, ACE-inhibitory, antimicrobial, or immunomodulatory properties. These activities depend on peptide sequence, molecular size, hydrophobicity, and resistance to gastrointestinal digestion. However, most peptide-specific mechanisms have been characterized *in vitro*, and their bioavailability, stability, and clinical relevance in humans remain incompletely understood.

Therefore, structure–activity relationships provide an important mechanistic rationale for the observed effects of whey proteins, but they also highlight a key limitation of the current evidence base: the biological activity of whey proteins cannot be generalized across all formulations. Isolates, concentrates, and hydrolysates may differ in protein purity, peptide profile, digestion kinetics, and functional properties. Future studies should therefore characterize whey protein form and peptide composition more precisely and evaluate whether these structural differences translate into clinically meaningful outcomes.

Several limitations of the current evidence base should be considered. First, the available studies are heterogeneous with respect to population characteristics, clinical setting, whey protein form, dose, duration of intervention, and outcome measures. Second, many mechanistic effects, particularly those related to DPP-IV inhibition, microbiota modulation, and anticancer activity, are derived mainly from *in vitro* or animal models. Third, clinical studies often evaluate whey protein as part of multi-nutrient formulations, which makes it difficult to isolate the independent effect of whey proteins from other components such as leucine, vitamin D, fiber, or prebiotics. Fourth, there is no clear consensus regarding optimal dosing strategies, including total daily intake, per-meal protein distribution, timing of administration, and formulation-specific effects. Finally, long-term safety and efficacy data in specific patient populations, including critically ill, oncological, renal, and metabolically compromised patients, remain limited.

These limitations do not negate the potential clinical relevance of whey proteins, but they indicate that their application in FSMPs should be guided by patient-specific needs, formulation characteristics, and the strength of available evidence rather than by generalized assumptions regarding superiority over other protein sources.

## 9 Conclusion

Whey proteins are high-quality, rapidly digested sources of indispensable amino acids, with a leucine-rich profile that supports postprandial muscle protein synthesis. Human studies also demonstrate acute effects on incretin secretion, insulin response, and postprandial glycemia. These findings provide the strongest basis for their use in clinically oriented nutritional formulations.

The evidence for additional effects is less mature. Cysteine availability and selected whey fractions provide plausible antioxidant and immunomodulatory mechanisms, whereas peptide-mediated DPP-IV inhibition, wound-healing effects, microbiota modulation, and the cytotoxicity of protein-lipid complexes are supported predominantly by mechanistic, *in vitro*, animal, or limited clinical data. These observations should not be interpreted as established therapeutic effects.

In FSMP development, whey protein form, dose, timing, co-formulated nutrients, processing history, tolerance, and the characteristics of the target population should be considered. No single whey preparation or dosing strategy can currently be recommended across all clinical groups. Adequately powered comparative trials are needed to define dose–response relationships, formulation-specific efficacy, clinically meaningful long-term outcomes, and safety in vulnerable patient populations.
